# An ALE Meta-Analysis on Investment Decision-Making

**DOI:** 10.3390/brainsci11030399

**Published:** 2021-03-21

**Authors:** Elena Ortiz-Teran, Ibai Diez, Joaquin Lopez-Pascual

**Affiliations:** 1 Facultad de Ciencias Jurídicas y Sociales, Universidad Rey Juan Carlos, 28032 Madrid, Spain; 2 Gordon Center for Medical Imaging, Department of Radiology, Massachusetts General Hospital, Harvard Medical School, Boston, MA 02115, USA; IDIEZPALACIO@mgh.harvard.edu; 3 Departamento de Economía de la Empresa, Facultad de Ciencias Jurídicas y Sociales, Universidad Rey Juan Carlos, 28032 Madrid, Spain; joaquin.lopez@urjc.es

**Keywords:** neuroeconomics, investor, stock, reward, risk, ventral striatum, anterior insula, amygdala, anterior cingulate cortex

## Abstract

It is claimed that investment decision-making should rely on rational analyses based on facts and not emotions. However, trying to make money out of market forecasts can trigger all types of emotional responses. As the question on how investors decide remains controversial, we carried out an activation likelihood estimation (ALE) meta-analysis using functional magnetic resonance imaging (fMRI) studies that have reported whole-brain analyses on subjects performing an investment task. We identified the ventral striatum, anterior insula, amygdala and anterior cingulate cortex as being involved in this decision-making process. These regions are limbic-related structures which respond to reward, risk and emotional conflict. Our findings support the notion that investment choices are emotional decisions that take into account market information, individual preferences and beliefs.

## 1. Introduction

“To invest successfully over a lifetime does not require a stratospheric IQ, unusual business insights, or inside information. What’s needed is a sound intellectual framework for making decisions and the ability to keep emotions from corroding that framework” [1] (p. ix). While there is no agreement on how emotions influence these decisions, there is a common understanding that expert investors are wired to weigh expected rewards and risks while making financial decisions. We believe that this kind of knowledge, acquired by a combination of formal training and on-the-job experience, is a trait of the brain.

What is interesting about this decision-making process is that it is all about expectations learned from experience, by constantly readjusting these predictions to the actual results [2]. A decision, whether risky or safe, begins with fluctuations of dopamine within the reward circuit [3]. However, to come to a decision, this process must integrate information coming from reward circuits as well as brain regions involved in cognition [4].

Although activity in dopaminergic brain areas has been shown to occur with both immediate and delayed rewards, in 2004, McClure et al. demonstrated that there are two distinct systems involved in choices between monetary reward options available at different moments in time [5]. On the one hand, decisions that entail immediate rewards involve the ventral striatum, the medial orbitofrontal cortex and the medial prefrontal cortex (mPFC) [5], all of which are activated by the receipt of rewards [6]. On the other hand, intertemporal choices of delayed rewards engage the lateral prefrontal cortex (lPFC) and the parietal cortex [5]. These latter areas of the brain are known to be implicated in the control of cognitive functions and goal-directed behavior, including the modulation of working memory information through rewards [7] and the representation of task-reward associations [8].

How, then, do investors choose between different types of rewards? The answer lies in a common scale of values. Thus far, neuroimaging studies in humans have highlighted the ventromedial prefrontal cortex/orbitofrontal cortex (vmPFC/OFC) as the key brain area for representing the subjective values of all reward types on a neural common scale and, to a much lesser extent, the ventral striatum [9]. The most feasible explanation is that the vmPFC computes these values by trading off costs and benefits from the amygdala and the ventral striatum, respectively [10]. Other areas, such as the anterior insula (AIns), engage with rewards by having a negative correlation with increasing anticipated monetary reward [11] as well as a positive correlation, mainly with the anterior cingulate cortex (ACC) when a decision conflict arises between options of competing value [12]. Therefore, the brain appears to be equipped with a unified valuation network to compare between rewards.

On the contrary, risk is determined by the probabilities of possible outcomes, which are estimated by individual perceptions based on previous experiences. How these evaluated probabilities influence investors’ decisions depends on the amount of information available (risk) or, rather, the information that is unknown to the investor (ambiguity). Several studies have associated activation between risk and ambiguity with distinct brain areas. Usually, risk activates the insula, the striatum and the parietal cortex, whereas ambiguity involves the lPFC, the mPFC, the cingulate cortex and the amygdala [13].

As opposed to rewards, the scientific community is still hesitant to draw strong conclusions about a unified neural system for evaluating decisions at all levels of uncertainty, despite the fact that the AIns is thought to encode changes in the amount of variability (risk) as well as risk prediction errors [14]. In 2005, Hsu et al. suggested a common neural circuit which was positively activated in the amygdala and the OFC and negatively in the striatum as uncertainty increased [15]. However, in 2019, FeldmanHall et al. ruled out these areas and stated that only the lPFC played a key role in processing high levels of uncertainty [16], despite its correlation with individual ambiguity preferences [17]. Nevertheless, all these areas are involved in the regulation of emotional responses, whether evoked consciously or automatically by the stimulus itself [18,19].

Psychological and neuroscientific research has emphasized that emotions play a role in decision-making, but it remains unclear how they influence risk processing and risk anticipation. Anticipatory effects in distinct neural circuits can impact financial choices [20]. For instance, risky and safe investments are predicted by ventral striatum and anterior insula activation, respectively [21]. It appears as if two parallel processes occur when a person makes an investment choice. On the emotional level, activity in the AIns assesses potential losses, while the thalamus can anticipate regret in the case of loss. On the cognitive level, the dorsomedial prefrontal cortex (dmPFC) evaluates risk by using the information provided by the AIns and the thalamus [22]. To decide, the parietal cortex and the dorsolateral prefrontal cortex (dlPFC) must combine the information about risk with the expected reward obtained from those areas [22].

It is not fully known which neuronal circuits drive investment decisions. An unbiased way to understand what leads a person to make some investments and not others is by using the coordinates reported from all task-related neuroimaging studies to determine brain activation while investing. Our aim is to summarize the structures specialized in responding to investment decision-making by conducting an activation likelihood estimation (ALE) meta-analysis from individual functional magnetic resonance imaging (fMRI) studies that have reported whole-brain analysis results during an investment task.

## 2. Methods

This meta-analysis was performed according to the PRISMA systematic reviews and meta-analyses guidelines [23].

### 2.1. Eligibility Criteria

We included studies that analyzed decision-making via investment tasks in healthy human adults without any other restrictions, such as language, publication date or text availability. Studies were eligible if they included fMRI as the only neuroimaging technique used and if they reported original data from whole-brain analysis results. We excluded all studies whose participants had suffered brain injuries, had any diseases or had disorders. We selected those studies that assessed investment decisions using financial assets. Furthermore, we restricted our selection to peer-reviewed articles.

### 2.2. Information Sources and Search

Studies were identified in the following electronic databases: WOS, PubMed and PsycINFO. The only filters used were species (humans) and age (adults). The search terms included the following: investment decision making; investment risk taking; investments; financial decisions; financial risk taking; investors; traders; trading (decisions); stock market; stock exchange; portfolio; market bubbles; financial bubbles; brain; and fMRI (see Appendix A for the search strategy using the WOS database).

### 2.3. Data Collection Process

Information was collected using a spreadsheet under the following headlines: authors, title, year of publication, number of participants, sex, age, stimuli, aim, behavioral results, brain activation and coordinates. If a study reported Talairach coordinates, we transformed them into Montreal Neurological Institute (MNI) space using the icbm2tal algorithm implemented in the GingerALE toolbox (https://www.brainmap.org/ale: available on 7 December 2020).

### 2.4. Meta-Analysis of Brain Activation Coordinates

GingerALE (version 3.0.2) was used to run the activation likelihood estimation (ALE) algorithm [24,25,26]. GingerALE meta-analytic software reveals concordant brain regions among the provided imaging studies, using random effects analysis to test the maximum activation probabilities against a null hypothesis of spatially independent activations. Cluster-level family-wise error thresholding at *p* < 0.01 was used to correct for multiple comparisons [27] due to its increased power and compromise between sensitivity and specificity. An initial cluster-forming threshold of *p* < 0.001 (uncorrected) was used, and 1000 permutations were applied.

### 2.5. Visualization

We used Caret v5.65 software to project the cortical results into a three-dimensional population-average landmark and surface (PALS-B12), using an enclosing voxel algorithm and fiducial mapping [28]. The subcortical slices were generated with in-house Matlab scripts. The ALE values of the meta-analysis were projected with a threshold of 0.0025 for visualizing the trend. Black borders were used to delineate the surviving regions to multiple comparisons.

## 3. Results

### 3.1. Study Selection

**Figure 1:**
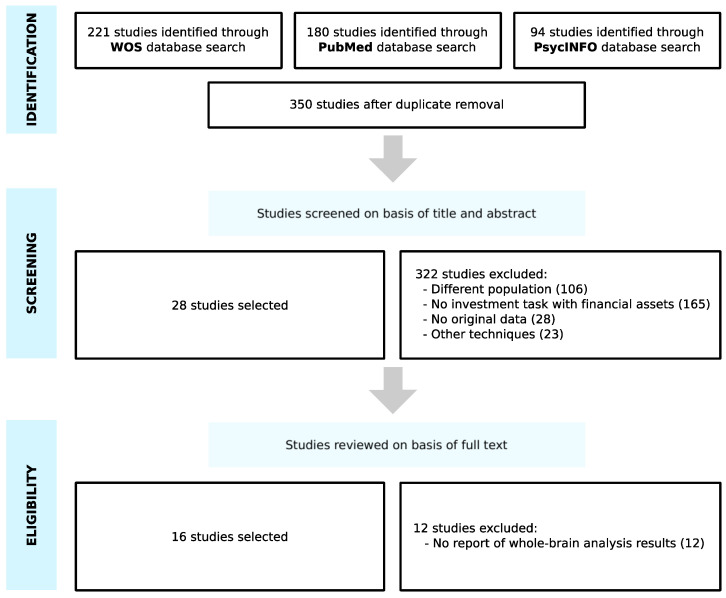
Search flow diagram adapted from PRISMA guidelines.

The search on the WOS, PubMed and PsycINFO databases was conducted from October 2020 to November 2020 and provided a total of 495 studies. Once duplicates had been removed, 350 studies were screened on the basis of titles and abstracts. We discarded 322 articles, as they did not meet the eligibility criteria; 106 studies belonged to a different population (participants with disorders, neurodegenerative diseases and brain injuries, healthy elderly people, adolescents and children), 165 studies did not involve an investment task with financial assets, 28 studies had no original data, and 23 studies used other techniques (electroencephalography, positron emission tomography or transcranial stimulation). Then, the full text of the remaining 28 studies were examined, and 12 studies were excluded due to the fact that they did not report whole-brain analysis results. As a result of the selection criteria, 16 studies were selected for the meta-analysis (Figure 1).

### 3.2. Study Characteristics

All articles employed some type of investment decision-making task. Four studies focused on the role that previous investments had on current decisions [29,30,31,32], while the rest concentrated on trading tasks.

The information presented was essentially market data. However, three studies also shared social information [33,34,35], and one included the responses from a computer partner as a control condition [36]. Only one study presented the stimuli under gain and loss domains [37], whereas three studies used market bubble conditions [38,39,40].

Eight studies focused on certain cognitive processes while choosing between investments, including sunk costs [30,31], disposition effects [41] and prediction errors [36,42,43].

Some studies included other behavioral tests with the same participants whose brains were being scanned. For example, questionnaires were conducted on the future time perspective [39], eye gaze [40] and self-assessment questions [37].

The included studies involved 594 healthy adults without any real-life experience in investing, except for one study [37]. Two studies included only males [37,40], and two did not report the sexes of the participants [29,35].

All sixteen studies were conducted with fMRI and were published between 2005 and 2018.

### 3.3. Study Results

Regarding studies with prior investments, it has been demonstrated that previous investments affect current decisions, making people more prone to continue investing. This is related to higher activation not only in the prefrontal and parietal cortices [29,30,31], but also in the anterior insula, due to its role in risky decision-making [29,32]. This latter brain area, along with the ventral striatum, has been repeatedly found to be active in tasks involving trading decisions [21,34,36,37,38,41,42,43].

Studies which included social information reported a higher activation in the ventral striatum when investors decided to follow herd buying behavior [34], as well as in the paracingulate cortex while forecasting price changes [35]. On the contrary, overweighting private information involved activity in the inferior frontal gyrus, the anterior insula [33] and the ACC to resolve social conflicts that arose from going against the group [34]. However, this does not apply if the information is non-human [34,36]. In the study on gain and loss domains, only the former, along with the anterior insula, could be related to real-life experience in trading stocks [37]. With respect to market bubble conditions, higher levels of nucleus accumbens (NAcc) and vmPFC activity [38,40] and dlPFC and inferior parietal lobule connectivity [39] indicate a propensity to ride bubbles and lose money.

Investment decisions can be affected by certain cognitive processes, such as higher sunk costs translating into more risk-taking behaviors in the lateral prefrontal and parietal cortices [30,31]; a higher disposition effect lowering ventral striatum activity because investors held onto losing assets longer [41]; fictive errors driving investment behavior through increased activity in the ventral striatum [36,43]; and decreased activity in anterior insula and anterior insula-amygdala connectivity when reappraisal strategies regulated negative feelings [42].

Studies with behavioral tests linked the estimation of future prices with activation in the inferior parietal lobule and future time perspective scores [39]; the ability to infer other investors´ intentions with signal changes in the dmPFC and eye gaze scores; and beliefs and preferences toward risk (risk optimism index and risk tolerance index) with activation in the anterior insula and real-life trading experience [37].

### 3.4. Meta-analysis of Brain Activation Results

**Figure 2:**
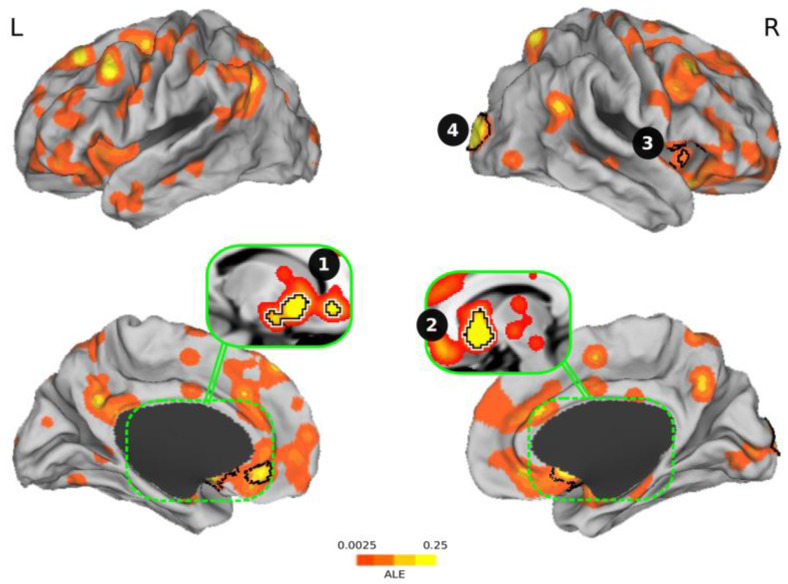
Overview of significant clusters resulting from the activation likelihood estimation (ALE) meta-analysis regarding investment decision-making. The four clusters found during risky and safe investments were (1) ventral striatum + amygdala + anterior cingulate cortex; (2) the ventral striatum; (3) the anterior insula; and (4) the occipital cortex. The clusters corrected for multiple comparisons are outlined in black. L = left, R = right.

Figure 2 and Table 1 display the results of the ALE meta-analysis we conducted. The four clusters we found were (1) ventral striatum + amygdala + anterior cingulate cortex; (2) the ventral striatum; (3) the anterior insula; and (4) the occipital cortex. Table 2 shows the characteristics of the studies included in the meta-analysis with the clusters reported by each study.

## 4. Discussion

It is not surprising that the first three clusters we found included areas of the brain that are closely related to the expectation of reward and risk, as investments are based on risk–return tradeoffs. The fourth cluster, the occipital cortex, is activated during investment decisions as market information gathered via computers is perceived through the visual pathway. Nevertheless, while investors consider the need to control their emotions in order to not interfere with their investment decisions, these same brain regions are also involved in emotions when assessing the value of environmental stimuli. Although areas such as the vmPFC/OFC did not survive multiple comparisons, Figure 2 shows that there was a tendency, which confirmed a role of these regions in investment decision-making, probably as a common scale of values. These results lead us to believe that investment choices are emotional decisions.

**Figure 3:**
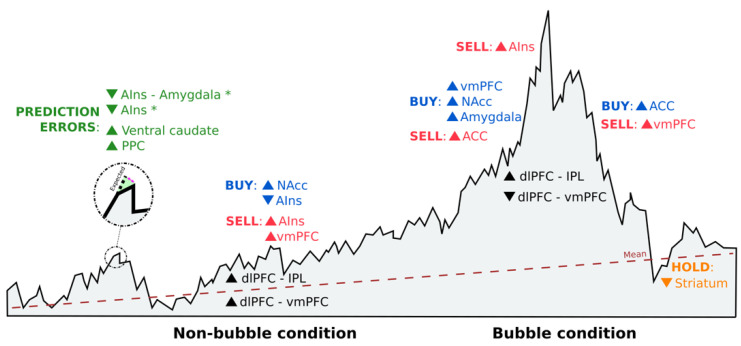
Schematic representation of brain activation reported in the ALE meta-analysis study during investment decision-making. Sell orders are shown in red, buy orders are in blue, and neither sell nor buy orders (hold) are in orange. Each order is accompanied by increased (▲) or decrease (▼) neural activity in certain brain areas. Prediction errors appear in green, where (*) indicates that reappraisal strategies were implemented. The location of each activation in the figure is based on the situation of the market, being under bubble or non-bubble conditions. Black indicates brain connectivity activation under both market conditions. AIns = anterior insula; PPC = posterior parietal cortex; NAcc = nucleus accumbens; vmPFC = ventromedial prefrontal cortex; ACC = anterior cingulate cortex; dlPFC = dorsolateral prefrontal cortex; and IPL = inferior parietal lobule.

Generally, perceived risks (AIns) and risk attitudes (lateral OFC) seemed to affect the value of the chosen investment (dlPFC and amygdala) [32]. Prior to deciding, value was correlated with mPFC, lPFC and posterior cingulate cortex activity [32], whereas distinct neural circuits involving the nucleus accumbens (NAcc) and the AIns seemed to promote risk-seeking and risk-averse choices, respectively [21] (Figure 3). However, while excessive activation in these areas may cause investment mistakes [21], reduced activation could lead to a learning process in which emotion regulation in fictive error signals (i.e., what might have happened) could guide valuation and choice [42] (Figure 3). These differences between actual returns and returns that could have been experienced if decisions had been diverse also drive investment behavior through significant ventral caudate and posterior parietal cortex activation [43] (Figure 3).

Seeking environmental validity for these results, we found an attempt to connect real-life financial behavior with brain activation during an investment task. Häusler et al. [37] demonstrated that choosing between a stock and a bond involved differences in brain activation in the AIns (Figure 3). Active stock traders showed lower AIns activation when choosing the risky option (stock) compared with those who did not trade in real life [37]. This may be due to individual differences in risk attitudes [29] and the way in which investors perceive risks [32]. Therefore, this difference was not based on cognitive abilities or financial constraints, but rather mediated by individuals´ preferences and beliefs about risky financial choices [37].

In financial markets, prices are determined by the interacting decisions of many investors. Inferring other agents´ intentions while making value judgments can lead to an increase in prices above their fundamental values, causing a market bubble. Under these conditions, social signals activate the paracingulate cortex [35] and the dmPFC [40], which affect value representations in the vmPFC [40], an area known to be associated with asset preferences [39,44]. This increased sensitivity in the vmPFC toward other investors´ intentions makes activity in this brain area a predictor of the tendency to ride bubbles [40] (Figure 3). Although investors can be predisposed to buying stocks in market bubbles, the vmPFC has also been found to correlate with cash holdings [39], probably due to its activation after monetary gains [21]. Nonetheless, functional connectivity in the vmPFC decreased as bubbles gave way to an increase in dlPFC–inferior parietal lobule (IPL) connectivity (Figure 3), since supportive information is required from the IPL to estimate future stock prices so that the dlPFC can decide [39].

Another brain area that is thought to track bubble magnitude, responding to both buying and selling outcomes, is the NAcc [38]. Increased NAcc activity is associated with lower returns [38], given the propensity to buy risky assets [21] in subsequent trading periods (Figure 3). By contrast, if the activity occurs in the AIns, it will serve as a risk detection signal that will result in higher earnings, due to a higher propensity to sell before the bubble reaches its peak [38] (Figure 3).

While there is no universally acknowledged explanation of how bubbles form, it is known that herd behavior often causes higher volatility in the stock markets [45], both up and down, as investors decide to get in or out at the same time. The reason for this behavior is that when faced with uncertainty, investors tend to imitate the actions of others. Activity in the ventral striatum is influenced by social information on other investors’ decisions, making one´s decision to buy or reject more in line with the stock bought or rejected by the herd [34], even when there is no advantage in doing so [36]. One feasible reason is that the striatum engages in prediction error signals, helping us learn the value of different options [36]. Aligning with the group also activates the amygdala [34], which may reflect a social learning process [46], and the middle cingulate cortex due to its sensitivity in identifying oneself with other investors´ behaviors [36]. However, if investors do not base their decisions on the behavior of others and act against the group, activity in the ACC increases to solve the social conflict that arises [34] (Figure 3). This would imply that investors update their beliefs by overweighting private information instead of social information, which results in higher activation of the inferior frontal gyrus–AIns and lower activation of the parietal-temporal cortex, areas known to be associated with risk and uncertainty [33].

It is evident that information can alter financial decision-making, especially if prior investments have been made. Throwing good money after bad in order to avoid realizing certain losses is a cognitive bias that investors are sensitive to. This effect, known as sunk costs, demonstrates that the amount of money that has already been allocated affects the decision of whether to continue an investment or not. When sunk costs are higher, activity in the lPFC, the parietal cortex [31], the amygdala and the ACC increases [30], given the desire to not appear wasteful [47]. At the same time, investors stop tracking the expected value of new investments to focus on previous investments to guide their current decisions so that the participation of the vmPFC and the NAcc in this decision process is considerably diminished, thereby making them prone to continue investing [30]. It has been found that there is a strong negative connectivity between the dlPFC and the vmPFC after an investment is made as a way to not waste resources while overriding the commonly expected value-based decision-making [30].

According to Kahneman and Tversky [48], aversion to loss realization is one of the reasons why investors fall into sunk costs, a fallacy that may strengthen the disposition effect. The disposition effect is a behavior which leads investors to “sell winners too early and ride losers too long” [49], based on an irrational belief in mean reversion [41]. There is a negative correlation between the disposition effect and ventral striatum activity related to rises in asset prices [41] (Figure 3). Expecting a return to the mean could be the reason behind an attenuated striatal response to upticks in value below the purchase price [41], given that dopamine neurons respond more strongly to unpredicted rewards [2].

There are three main limitations to this review. The first limitation comes from a small sample size, considering that neuroeconomics is still a new field and most of the studies to date have focused on specific regions of interest instead of whole-brain analysis. The second limitation arises from the different stimuli and aims used in all investment decision-making tasks. For example, some studies have presented stimuli in a moving display or used live trading, which resemble more closely what happens in real-life financial decisions, as opposed to static stimuli trying to evoke actual dynamic markets. The third limitation derives from the lack of active stock traders as participants, except for the study by Häusler et al. [37], given that environmental factors can shape individual financial decisions.

## 5. Conclusions

Investment decisions can overwhelm the brain. Trying to make sense of all information that financial markets convey while listening to one´s emotions without being overridden by them involves a coordinated effort of several brain areas in order to reach a decision. Since the question of how investors make decisions has not yet been fully uncovered, the aim of this meta-analysis is to determine the convergence of brain regions necessary for this complex decision-making process. Based on our ALE meta-analysis results, investment decisions involve limbic areas that ponder reward vs. risk, as investment portfolios are built on trying to achieve an optimal balance between return and risk. Emotions toward these two concepts, and the emotional conflicts that can arise while prioritizing among them, are an influential factor that guide this decision process. As Benjamin Graham has noted, “individuals who cannot master their emotions are ill-suited to profit from the investment process.” Despite the four clusters found, we believe that investment decisions are not limited to those areas alone. In the stock market, aspects such as when to buy or sell, the market conditions or even the way in which other investors behave can affect whether an investment will result in being profitable or not. As Warren Buffet once advised, “be fearful when others are greedy and greedy when others are fearful.” Every investor knows how to be fearful and greedy, but what they truly need to discover is when the right time to be one or the other is. The same investment behavior and the same brain activation could lead to different yields depending on the moment. Determining the role of a specific brain area in this decision-making process is indeed a complicated endeavor. Herein lies the difficulty in understanding how investors make decisions. Given the scarce literature, future studies should continue addressing this decision-making process while including whole-brain analysis in their methods.

## Figures and Tables

**Table 1:** Significant clusters of the meta-analysis surviving to multiple comparisons.

Cluster #	Brain Areas	Size (mm^3^)	Center Coordinate	Peak Coordinate	ALE	*P*	Z
1	Ventral striatum + amygdala + ACC ^1^	6360	(−11.8, 13.3, −7.8)	(−10, 16, −4)	0.0439	*p* < 0.0001	6.59
2	Ventral striatum	3976	(11.2, 13.1, −5.8)	(10, 14, −6)	0.0748	*p* < 0.0001	9.44
3	Anterior insula	2048	(22.6, −95.3, 8.8)	(22, −96, 8)	0.0611	*p* < 0.0001	8.24
4	Occipital cortex	1544	(49.1, 18.6, −3.9)	(54, 16, −4)	0.0303	*p* < 0.0001	5.11

^1^ ACC = anterior cingulate cortex.

**Table 2:** Investment decision-making studies included in the meta-analysis.

References	Stimuli	Brain and Behavioral Results	Cluster #
Kuhnen et al., 2005	Two stocks (one good and the other bad) and a bond	Anticipatory nucleus accumbens activity preceded risky choices, and excessive levels of activation led to risk-seeking mistakes. Anticipatory anterior insula activity preceded riskless choices, and excessive levels of activation led to risk-aversion mistakes.	1 2
Lohrenz et al., 2007	Market information in live and not live conditions, gains and losses, portfolio value and percentage already invested	Higher levels of ventral caudate activity correlated with fictive error signals, driving investment behavior.	1 2 3
Mohr et al., 2009	Streams of 10 past returns from an investment	Risk and value are represented in the brain during investment decisions in discrete (simple gambles) and continuous distributions (stocks). Risk–return models support the correlation between risk and anterior insula activation.	1 4
Bruguier et al., 2010	Replay of market experiment sessions (order and trade flow) with and without insiders	Theory of mind is involved in forecasting price changes in markets with insiders and related to increased activation in the paracingulate cortex.	
Burke et al., 2010	Stock information and social information (four human faces or four chimpanzee faces)	Higher levels of ventral striatum activity correlated with the participants´ likelihood to follow herd behavior, especially in the number of buying decisions. Going against the group involves activity in the anterior cingulate cortex to resolve the conflict.	1 2
Brooks et al., 2012	Purchase prices and asset prices (random walk)	The irrational belief in mean reversion better explains the disposition effect. Participants with a large disposition effect exhibited lower levels of ventral striatum activity in response to upticks in value when the asset price was below the purchase price.	1 2 3
De Martino et al., 2013	Portfolio value and trading prices (asks and bids) in bubble and non-bubble markets	The evaluation of social signals in dorsomedial prefrontal cortex activity affects value representations in the ventromedial prefrontal cortex. Higher levels of ventromedial prefrontal cortex activity predict an investor’s propensity to ride bubbles and, therefore, lose money.	
Zeng et al., 2013	Amounts already invested in a company´s project where sunk costs and incremental costs are manipulated	Higher levels of lateral frontal and parietal cortex activity are related to higher sunk costs and more risk-taking behavior. Higher levels of striatum and medial prefrontal cortex activity are linked to smaller incremental costs and continued investing.	
Lohrenz et al., 2013	Market data and social information (other players´ bets)	Interpersonal fictive errors guide behavior and highly correlate with striatum activity.	1 2
Ogawa et al., 2014	Stock and asset information in a virtual stock exchange with two non-bubble stocks and one bubble stock	In market bubbles, brain networks switch toward dorsolateral prefrontal cortex and inferior parietal lobule connectivity, in which buying decisions are made in the former based on the information gathered by the latter region. Cash holdings were positively correlated with activation in the ventromedial prefrontal cortex, while trading during large price fluctuations were associated with superior parietal lobule activity.	
Smith et al., 2014	Trading prices of risk-free and risky assets (stocks) in markets where endogenous bubbles are formed and crash	Higher levels of nucleus accumbens activity are associated with buying decisions, lower earnings, and increased likelihood of a crash. Higher levels of anterior insula activity are correlated with selling decisions before the price peak and higher earnings.	1 2
Haller et al., 2014	Project costs and success probabilities	Higher levels of dorsolateral prefrontal cortex and lower levels of ventromedial prefrontal cortex activity are related to higher sunk costs and being prone to continue investing in previous investments.	1 2
Gu et al., 2014	Market prices where choices are made under two conditions: regulate and attend	Only fictive errors are susceptible to reappraisal strategies by changes in activation in anterior insula and anterior insula–amygdala connectivity, modulating subjective feelings that affect behavior directly.	1 3
Huber et al., 2015	Two stocks with social (decisions made by two fictitious traders) and private information (personal recommendation from a rating agency)	Higher levels of inferior frontal gyrus/anterior insula activity and lower levels of parietal-temporal cortex activity are correlated with overweighting private information, which can influence the probability in the formation of informational cascades.	2
Majer et al., 2016	Past returns of investments and investment choices with fixed or risky returns	Higher levels of anterior insula and dorsomedial prefrontal cortex activity correlated with risk and decision-making.	
Häusler et al., 2018	Stocks (risky option) and bonds (non-risky option) in gain and loss domains	Lower levels of anterior insula activity are connected to risky decisions in real-life stock traders. These choices are based on personal beliefs about risky choices and the willingness to bear risk.	1 3

## Data Availability

Not applicable.

## Code Availability

All codes are available for the research community from the corresponding author upon request.

## Appendix A

Search strategy using the WOS database:

      Financial decisions AND fmri

            (Search results: 94)

      Financial risk taking AND fmri

            (Search results: 29)

      Investments AND fmri

            (Search results: 75)

      Stock market AND fmri

            (Search results: 8)

      Trading AND investors AND brain

            (Search results: 12)

      Market bubbles AND fmri

            (Search results: 3)
